# Autologous bone marrow derived mesenchymal stromal cell therapy in combination with everolimus to preserve renal structure and function in renal transplant recipients

**DOI:** 10.1186/s12967-014-0331-x

**Published:** 2014-12-10

**Authors:** Marlies EJ Reinders, Jonna R Bank, Geertje J Dreyer, Helene Roelofs, Sebastian Heidt, Dave L Roelen, Volkert AL Huurman, Jan Lindeman, Cees van Kooten, Frans HJ Claas, Wim E Fibbe, Ton J Rabelink, Johan W de Fijter

**Affiliations:** Department of Nephrology, Leiden University Medical Center, Albinusdreef 2, 2300 RC Leiden, the Netherlands; Einthoven Laboratory for Experimental Vascular Medicine, Leiden University Medical Center, Albinusdreef 2, 2300 RC Leiden, the Netherlands; Department of Immuno-haematology and blood transfusion, Leiden University Medical Center, Albinusdreef 2, 2300 RC Leiden, the Netherlands; Department of Surgery, Leiden University Medical Center, Albinusdreef 2, 2300 RC Leiden, the Netherlands

**Keywords:** Mesenchymal stromal cells, Renal transplantation, Fibrosis, Immune modulation, Repair

## Abstract

**Background:**

Kidney transplantation has improved survival and quality of life for patients with end-stage renal disease. Despite excellent short-term results due to better and more potent immunosuppressive drugs, long-term survival of transplanted kidneys has not improved accordingly in the last decades. Consequently there is a strong interest in immunosuppressive regimens that maintain efficacy for the prevention of rejection, whilst preserving renal structure and function. In this respect the infusion of mesenchymal stromal cells (MSCs) may be an interesting immune suppressive strategy. MSCs have immune suppressive properties and actively contribute to tissue repair. In experimental animal studies the combination of mammalian target of rapamycin (mTOR) inhibitor and MSCs was shown to attenuate allo immune responses and to promote allograft tolerance. The current study will test the hypothesis that MSC treatment, in combination with the mTOR inhibitor everolimus, facilitates tacrolimus withdrawal, reduces fibrosis and decreases the incidence of opportunistic infections compared to standard tacrolimus dose.

**Methods/design:**

70 renal allograft recipients, 18–75 years old, will be included in this Phase II, open label, randomized, non-blinded, prospective, single centre clinical study. Patients in the MSC treated group will receive two doses of autologous bone marrow derived MSCs IV (target 1,5x10^6^, Range 1-2x10^6^ million MSCs per/kg body weight), 7 days apart, 6 and 7 weeks transplantation in combination with everolimus and prednisolone. At the time of the second MSC infusion tacrolimus will be reduced to 50% and completely withdrawn 1 week later. Patients in the control group will receive everolimus, prednisolone and standard dose tacrolimus. The primary end point is to compare fibrosis by quantitative Sirius Red scoring of MSC treated and untreated groups at 6 months compared to 4 weeks post-transplant. Secondary end points include: composite end point efficacy failure (Biopsy Proven Acute Rejection, graft loss or death); renal function and proteinuria; opportunistic infections; immune monitoring and “subclinical” cardiovascular disease groups by assessing echocardiography in the different treatment groups.

**Discussion:**

This study will provide information whether MSCs in combination with everolimus can be used for tacrolimus withdrawal, and whether this strategy leads to preservation of renal structure and function in renal recipients.

**Trial registration:**

NCT02057965.

## Background

Kidney transplantation has improved life expectancy and quality of life for patients with end-stage renal failure. However, despite the impressive improvements in short-term outcome parameters due to better and more potent immunosuppressive drugs, the long-term survival of renal allografts has changed little during the past decades [1]. A number of factors, such as quality of the graft, ischemia/reperfusion (I/R) injury, ongoing cellular and humoral alloreactivity and/ or calcineurin inhibitors (CNI) may adversely affect renal structure causing early tubular atrophy and interstitial fibrosis (IF/TA). CNI have been the cornerstone of immunosuppressive therapy for many years, due to their efficacy in preventing acute rejection. However, CNI have nephrotoxic side effects that can directly contribute to renal dysfunction and compromise long-term outcomes. Consequently, there is a clear need for immunosuppressive regimens that maintain efficacy for the prevention of rejection, whilst preserving renal function and structure. The immune regulatory properties of mesenchymal stromal cells (MSCs) in both cellular and antibody mediated inflammatory models have highlighted their potential to regulate the immune response after solid organ transplantation [2]. In addition, MSCs have been shown to ameliorate I/R injury and to exert reparative properties. The administration of MSCs might be an optimal strategy to facilitate CNI withdrawal and to minimize immune suppression. In addition, both MSCs and everolimus might improve cardiovascular status and this strategy might be an opportunity to reduce the toll of cardiovascular disease following kidney transplantation.

### Mesenchymal stromal cells

MSCs are multi potent cells that can be isolated from the bone marrow (BM) and many other sources. There is currently not a single marker that can distinguish MSCs from other cell types. Functional characterization of MSCs relies primarily on their ability to adhere to plastic and their differentiation potential. The International Society of Cellular Therapy stated that MSCs should bear at least the stromal markers CD73, CD90 and CD105, in addition to the absence of the hematopoietic markers CD14, CD34 and CD45 [3,4]. Important for their possible clinical application is that MSCs are easily isolated as they adhere to plastic and are capable of substantial proliferation and expansion in culture [5]. Another advantage is that MSCs can be cryopreserved with no loss of phenotype or differentiation potential [6].

Several studies suggest that MSCs may play a role in modulation of immune responses as extensively reviewed [7,8]. Indeed, MSCs can down regulate many immune effector functions and have also been found to induce regulatory cells [9,10]. These immune modulatory properties make MSCs especially attractive for potential use in treating disease driven by an immune response, including transplant rejection [7,8,11,12]. In addition, MSCs have been shown to improve tissue damage in response to injury. In animal models, MSC administration decreased fibrosis in the heart [13], and other organs such as the lung, liver and kidney [14-18]. Several cytokines have been shown to mediate the anti-fibrotic properties, including BMP-7 [18] and HGF [19]. In addition, various models have shown reparative properties in cardiovascular disease [20]. This is of importance for transplant recipients since cardiovascular disease causes significant morbidity and mortality in these patients. Different studies have suggested that the capacity of MSCs to produce paracrine factors plays a prominent role in affecting tissue repair and immune modulation [11,21].

### Mesenchymal stromal cells and solid organ transplantation

Beneficial immune modulatory effects of MSCs have been shown in experimental models of allo immune disorders. In the case of solid organ transplantation, the use of MSCs for several indications have been tested, including treatment of I/R injury, prevention of IF/TA, minimization of immune suppression and reversal or stabilization of chronic transplant inflammation and fibrosis as recently reviewed [22]. Ongoing immune injury to the graft may be caused by cellular and/or humoral mechanisms accompanied by de novo donor specific antibody (DSA) formation [23-25]. The importance of these de novo DSA as a major cause of allograft failure in the long term has recently been confirmed in numerous studies [23,25]. Importantly, it was shown that DSAs with the ability to activate complement, as determined by binding of C1q, are associated with greater risk of acute rejection and allograft loss [24]. By their immunosuppressive properties, MSCs may possibly serve an important role to control lymphocyte and antibody induced damage to the kidney.

In a rat heart transplantation model donor MSCs suppressed allogeneic T-cell responses *in vitro* and *in vivo* and intravenous administration of MSCs prolonged the survival of transplanted hearts, possibly by induction of allograft tolerance through changing the Th1/Th2 balance [26]. Interestingly, a recent study showed that heart grafts, which were tolerized through third-party multipotent adult progenitor cells, could be retransplanted to secondary hosts without immunosuppression [27]. In a study by Casiraghi et al., pre transplant infusion of MSCs prolonged the survival of semi-allogeneic (B6C3 in B6) murine heart transplants through the generation of regulatory T cells. A single recipient-derived MSC infusion given peri transplant was marginally effective, and a single MSC dose given one day after transplantation was not effective at all, emphasizing the importance of timing. The same group investigated the optimal timing for MSC infusion to promote immune tolerance in a murine kidney transplant model [28]. Pre-transplant MSC infusion induced a significant prolongation of kidney graft survival by inducing T regs [28], however post-transplant infusion caused premature graft dysfunction and failed to prolong graft survival. These results suggest that the inflammatory milieu is of importance for the mechanistic function of MSCs.

Two experimental studies are of particular interest for our clinical protocol. After kidney transplantation, Franquesa et al. observed a therapeutic effect of MSCs attenuating the progression of IF/TA when this process was already in progress [17]. Besides a reduction in IF/TA, MSC-treated animals demonstrated fewer macrophages infiltrating the parenchyma, lowered expression of inflammatory cytokines in combination with increased expression of anti-inflammatory factors [17]. In another experimental model, the combination of mTOR inhibitor and MSCs was shown to attenuate alloimmune responses and to promote allograft tolerance in heart transplants [29]. Indeed, combination therapy of MSCs and low-dose mTOR inhibitor rapamycin achieved long-term heart graft survival (>100 days) with normal histology. The treated recipients did accept donor skin grafts but rejected third-party skin grafts, indicating the establishment of donor specific tolerance. Tolerant recipients exhibited neither intragraft nor circulating DSA, but demonstrated significantly higher frequencies of both tolerogenic dendritic cells (Tol-DCs) and CD4^+^CD25^+^Foxp3^+^T cells in the spleens [29].

A limited number of clinical studies have investigated the role of MSCs in the transplant setting. Two studies have focused on the role of MSCs in the early induction phase. In a pilot study of safety and clinical feasibility, autologous MSCs were tested in renal transplant recipients. MSC infusion was shown to be feasible, allowing an increase of T-reg in the peripheral blood and control of memory CD8^+^T cell function [30]. In these patients, timing of the infusion seemed of major importance. Administration of MSC in the early phase after transplantation negatively affected kidney graft function, which was not the case when MSCs were administered before transplantation [31]. In a trial among 159 patients undergoing renal transplantation, the use of autologous MSCs compared with anti-IL-2 receptor antibody induction therapy resulted in lower incidence of acute rejection, decreased risk of opportunistic infection and better estimated renal function at 1 year [32]. Moreover, in a clinical pilot study allogeneic MSCs were administered in 6 renal transplant recipients. Allogeneic donor derived MSCs combined with low dose tacrolimus was safe and prevented acute rejection after renal transplantation [33], however immune monitoring was not performed in this study.

In our phase 1 clinical study safety and feasibility of autologous MSC therapy was tested in HLA-DR mismatched patients with subclinical rejection (SCR) in their renal biopsy at 4 or 24 weeks after renal transplantation [34,35]. In total 6 patients received MSC infusion which was feasible and well tolerated without adverse events related to the treatment itself. In addition, initial results suggested immune suppression after MSC therapy. All patients that received MSCs demonstrated a profound reduction in proliferation of patient peripheral blood mononuclear cells (PBMCs) 12 weeks after MSC infusion upon stimulation with donor specific PBMCs, while the response to third party PBMCs was more variable. Three patients developed opportunistic viral infections, which might have been related to the MSC treatment. In 2 patients with allograft rejection the infiltrate had disappeared after the MSC infusion. In addition, in patients diagnosed with IF/TA, the areas of IF/TA disappeared after treatment with MSCs, indicating that MSCs might play a role as antifibrotic and reparative treatment, which is one of the objectives in the current study. Effects of MSC therapy in cardiovascular disease have not been studied in previous clinical trials in renal transplant recipients.

## Methods and design

### Objectives and endpoints

The primary end point of the current study is the level of fibrosis, as determined by quantitative Sirius Red (SR) scoring of biopsies of MSC treated and untreated groups at 6 months compared to 4 weeks post transplantation. Other endpoints include: composite endpoint efficacy failure (Biopsy Proven Acute Rejection (BPAR), graft loss) at 6 months; renal function measured by eGFR (MDRD formula and iohexol clearance) and proteinuria at 6 months; cytomegalovirus (CMV), BK infection (viremia, disease and -syndrome) and other opportunistic infections; adverse events; the presence of DSA and other phenotypical and functional aspects of the donor specific immune response; to compare the progression of “subclinical” cardiovascular disease in the different treatment groups by assessing echocardiography.

### Study design

The current trial is a 6-month, randomized, open-label, non-blinded, prospective, single-center study of efficacy and safety comparing concentration-controlled everolimus and MSCs to everolimus with standard tacrolimus. The protocol has been authorized by the Dutch Government (CCMO) and by the Committee Medical Ethics of the LUMC (Leiden University Medical Center).

In total, 70 de novo renal recipients, 18–75 years of age will be recruited from the transplant clinics of the LUMC and enrolled into the study if they meet the eligibility criteria.

#### Inclusion criteria

For entry in the study, the following criteria must be met:

1. Female or male, aged between 18 and 75 years.
2. Subject is willing to participate in the study, must be able to give informed consent and the consent must be obtained prior to any study procedure.
3. Recipients of a first kidney graft from a deceased, living-unrelated or non-HLA identical living related donor > 50 years of age.
4. Panel Reactive Antibodies (PRA) ≤ 10%.
5. Patients must be able to adhere to the study visit schedule and protocol requirements.
6. If female and of child-bearing age, subject must be non-pregnant, non-breastfeeding, and use adequate contraception.

#### Exclusion criteria

1. Double organ transplant recipient.
2. Biopsy proven acute rejection (according to the Banff criteria) in the first 6 weeks after transplantation.
3. Patients with evidence of active infection or abscesses (with the exception of an uncomplicated urinary tract infection) before MSC infusion.
4. Patients suffering from hepatic failure.
5. Patients suffering from an active autoimmune disease.
6. Patients who have had a previous BM transplant.
7. A psychiatric, addictive or any disorder that compromises ability to give truly informed consent for participation in this study.
8. Use of any investigational drug after transplantation.
9. Documented HIV infection, active hepatitis B, hepatitis C or TB according to current transplantation inclusion criteria.
10. Subjects who currently an active opportunistic infection at the time of MSC infusion (e.g., herpes zoster [shingles], CMV, *Pneumocystis carinii* (PCP), aspergillosis, histoplasmosis, or mycobacteria other than TB, BK) after transplantation.
11. Malignancy (including lymphoproliferative disease) within the past 2-5 years (except for squamous or basal cell carcinoma of the skin that has been treated with no evidence of recurrence) according to current transplantation inclusion criteria.
12. Known recent substance abuse (drug or alcohol).
13. Contraindications to undergo a BM biopsy.
14. Patients who are recipients of ABO incompatible transplants.
15. Cold ischemia time >30 hrs.
16. Patients with severe total hypercholesterolemia (>7.5 mmol/L) or total hypertriglyceridemia (>5.6 mmol/L) (patients on lipid lowering treatment with controlled hyperlipidemia are acceptable).

**Figure 1:**
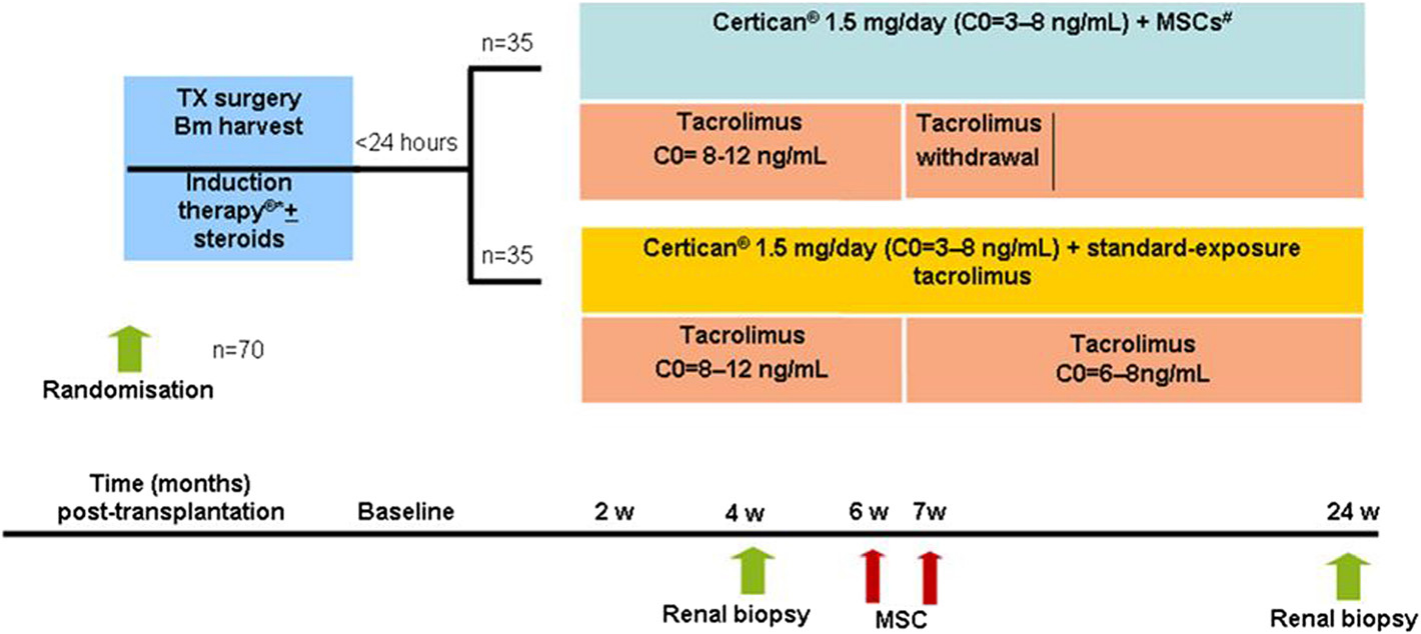
**Study scheme.** In total 70 patients will be included in the study, 18–75 years old. Patients will be randomised prior to transplantation. Thirty five of these patients will be included in the Certican®/MSC group and 35 patients in the Certican®/standard dose tacrolimus group. All patients will receive steroids (100 mg at day 1 to 3, 50 mg at day 4, 20 mg at day 5 to 15, 15 mg at day 15 to 21, and 10 mg after day 22) and induction treatment with alemtuzumab at day 0 and 1 (15 mg subcutaneously)*. Certican® dose will be 1.5 mg b.i.d. with trough levels between 3 and 8 ng/ml. Tacrolimus will be started orally 3 h before surgery (initial dose 2x5 mg ). In the first 6 weeks target trough levels are aimed at 10 ng/ml (range 8 to 12 ng/ml) for tacrolimus and thereafter 6–8 ng/ml. In the MSC treated group, BM will be harvested just prior to the renal transplantation and MSCs will be cultured in the GMP laboratory. Patients will receive 2 doses of a target of 1,5x10^6^ autologous BM MSCs per/kg body weight IV (range 1-2x10^6^) 7 days apart, 6 and 7 weeks after transplantation. The dose of tacrolimus will be reduced to 50% at the time of the second MSC infusion and completely withdrawn 1 week later. Patients will receive at that time point 15 mg of prednisolone. In all patients a renal biopsy will be performed at 4 weeks and at 6 months and scored according to the Banff criteria.

Thirty five of the patients will be included in the everolimus/MSC group and 35 patients in the everolimus/standard dose tacrolimus group (Figure 1, study scheme). All patients will receive steroids and induction treatment with alemtuzumab at day 0 and day 1 (15 mg subcutaneously). BM will be harvested just prior to the renal transplantation only from patients randomised to the MSC treatment group. MSCs will be cultured in the GMP laboratory, as previously described [34]. Patients will receive 2 doses of a target of 1,5×10^6^ MSCs per/kg body weight (range 1-2×10^6^) at weeks 6 and 7 after transplantation. The dose of tacrolimus will be reduced to 50% at the time of the second MSC infusion and completely withdrawn 1 week later. At that time point the patient will receive 15 mg prednisolon. The expected period of patient accrual is approximately 24 months. All study procedures will take place at the LUMC.

### Study procedures

Subjects will be seen in accordance with the assessment schedule listed below (Table 1).

**Table 1:** **Assessment schedule**

**Baseline**		**Tx date**	**Protocol renal biopsy**	**First MSC infusion**	**Second MSC infusion**								
**Week**		**D0**	**W4 a 5**	**W6**	**W7**	**W8**	**W9**	**W10**	**W12**	**W14**	**W16**	**W20**	**W24**
BM harvesting		X											
MSC expansion, generating		X											
Informed consent	X												
Medical history	X	X	X	X	X	X	X	X	X	X	X	X	X
Concomitant medication	X	X	X	X	X	X	X	X	X	X	X	X	X
Transplantation information	X	X	X	X	X	X	X	X	X	X	X	X	X
Physical examination	X	X	X	X	X	X	X	X	X	X	X	X	X
Routine lab	X	X	X	X	X	X	X	X	X	X	X	X	X
Viral load CMV and BK		X	X	X		X		X		X		X	X
Urinalysis	X	X	X	X	X	X	X	X	X	X	X	X	X
MSC infusion				X	X								
Renal biopsy		X	X										X
Sera for storage		X	X			X			X				X
Iohexol clearance			X										X
Blood for immune monitoring		X	X	X		X			X				X
DSA		X	X#						X				X
Safety assessment	X	X	X	X	X	X	X	X	X	X	X	X	X
Echo cardiography and pulse wave velocity			X										X

Follow up study visits are planned at baseline, day of transplantation, during the renal biopsy (week 4 a 5), during the first and second MSC infusion at week 6 and 7, week 8, 9, 10, 12, 14, 16, 20, 24 after transplantation.

### Isolation of bone marrow and infusion of MSCs

BM will be aspirated from the posterior iliac crest of all patients under general anesthesia during the renal transplantation. A total volume of 100 a 120 ml will be harvested. The processing of the cells will take place at the GMP Stem Cell Laboratory Facility of the LUMC.

In consented patients, a clinical re-evaluation will be undertaken before the planned infusion of MSCs. A target of 1,5×10^6^ MSCs per/kg body weight (range 1-2×10^6^) will be infused within 30 minutes as indicated. Actual doses of MSCs administered will be documented for each patient. Close monitoring of vital signs (temperature, pulse, respiratory rate, blood pressure, and oxygen saturation) will be measured and documented before MSC infusion, every 15 minutes in the first hour and every 30 minutes in the second hour after infusion.

### Data collection

Patients enrolled in this study will undergo standard pre-transplant work-up, which consists of baseline clinical data (demographics, medical history, current medication, previous blood transfusions, percentage of panel reactive antibodies, infection status (see below), physical examination, laboratory examinations, urinalysis, electrocardiogram, chest X-ray, infection screening). For women, the menopausal status will be recorded. The pregnancy test will be done prior to transplantation (according to our standard transplantation criteria). Results must be available and negative prior to administration.

Intraoperative data (warm and cold ischemia time, blood loss) and background information of the donor (age, gender, race, height, weight, type of allograft (living related or unrelated), infection status, serum creatinine) and HLA (mis)match will also be documented. All immunosuppressive and other drugs used and dosages administered will be recorded during the study.

### (Opportunistic) infections

HBsAg, Hepatitis C and HIV evaluated for screening will be performed standard before transplantation. Earlier tests, within 6 months prior to baseline are acceptable. CMV (PCR-positive), EBV (PCR-positive), BK-viruria in urine samples and BK-viremia in blood samples (RT-PCR) will be measured at baseline, week 4, 6, 7, 8, 10, 14, 20 and 24. Other infections (including urinary tract infections, pulmonary infections, herpes simplex) will be recorded as well. Patients are treated routinely with valganciclovir profylaxis per os for 6 months except for a CMV negative donor recipient status. In addition, all patients receive 6 months of cotrimoxazole prophylaxis against pneumocystis jirovecii pneumonia.

### Renal function

Glomerular filtration rate (GFR calculation) will be used to measure the renal function. The following abbreviated MDRD formula will be used for GFR estimation eGFR [mL/min/1.73 m2] = 186.3x SCr-1.154 × Age-0.203 × (0.742 if female) × (1.21 if black). In addition we will measure renal function with iohexol clearance at week 4 and 24. The iohexol clearance will be performed at the day of the renal biopsies.

### Fibrosis scoring by renal biopsy

A standard renal protocol biopsy is performed prior to the transplantation and at 6 months after transplantation. At 4 weeks after transplantation a study biopsy is taken to assess the renal histology before MSC infusion. Biopsies are scored according to the Banff criteria and processed for immunohistochemistry (HE staining; staining for CD3, CD4, CD68, FOXp3, C4d and CD20). In addition, the amount of cortical collagen (SR-positive area) will be measured and finally expressed as the percentage of the total analyzed cortical surface. Moreover, changes in mRNA expression of pro- and anti-fibrotic genes (including FGFb and fibronectin) in renal biopsies taken before and after MSC infusion through real-time quantitative PCR will be performed.

### Cardiovascular follow-up

Left ventricular internal dimension and wall thickness will be measured at end-diastole and at end-systole according to recommendations of American Society of Echocardiography. End diastolic left ventricular septal and posterior wall thicknesses and internal dimensions will be used to calculate left ventricular mass. Echocardiography will be performed at week 4 and week 24.

### Immune modulating capacities before and after MSC infusions

DSAs will be measured at baseline (before transplantation), time of renal biopsy, 12 weeks and 6 months after transplantation and every time a for-cause allograft biopsy is performed. For immunological monitoring, we will collect sera and PBMCs at different times post transplantation as described. Phenotypical analyses of the different leucocyte subpopulations will be performed on basis of the immune panels developed and validated for the One Study [36].

In addition mixed lymphocyte reaction assays of recipient’s PBMCs will be performed sequentially with the use of frozen cells obtained before transplantation to compare responses to the donor cells before and after transplantation [37]. Supernatants of these MLR cultures will be collected for analyses of cytokine profiles. Direct cross-matches of recipient sera and donor lymphocytes will be done by complement dependent cytotoxicity (CDC) and flow-cytometric assays. PBMCs will be stimulated using CD3/CD28 and analyzed for TH1 (i.e. interleukin-2 and interferon-γ), TH2 (IL-10 and IL-4) and inflammatory cytokines (i.e. tumor necrosis factor-α, TGF-β, IL-1 and IL-6).

### Data safety monitoring committee

The DSMB will monitor the safety of subjects. The DSMB consists of two independent physicians and one biostatistician. The DSMB will meet at least after inclusion of 20 and 50 subjects. They will judge on the rate of rejections and serious adverse events (SAEs) in the study. We regard a 30% rejection rate and over to be unacceptable; this will lead to study termination. The DSMB will review all serious adverse events unblinded and determine, based on a careful consideration of the events, whether the SAE is most likely related to MSCs. This review will take all aspects into account, including onset of the SAE relative to MSC infusions, other potential causes for the SAE such as concomitant medication and underlying conditions, and other previous adverse events observed over the course of the study. Only SAE that were assessed by the DSMB as most likely related to MSC will be taken into account for the decision to proceed with the study. The DSMB has the right to terminate the study.

### Sample size calculation

In our study a sample size of 25 in each group (or 50 in total) will have a 80% power to detect a difference in mean percentages of fibrosis of at least 25% using a two group t-test with a 0.05 two-sided significance level (alpha). In this calculation we used the following assumptions: we expect the control group (no MSC’s) to have a mean percentage of fibrosis of 18% at 6 months [38-43]. We assume that both groups show equal variability in measured fibrosis and that the common standard deviation is 5% [40]. A mean percentage of 14% in MSC group (25% less fibrosis) is considered a clinically relevant difference. We anticipate that 70% of included patients will have valid measurements (withdrawal included). We therefore plan to include 70 patients in total, 35 in the MSC group and 35 in the control group.

### Risk-benefit assessment

The high prevalence of nephrotoxicity suggests that CNI are unsuitable as long-term immunosuppressive agents for kidney transplantation and MSCs might offer an alternative treatment modality with the aim to inhibit fibrosis and to prolong allograft survival. In previous studies in transplant recipients, MSC therapy was shown to be feasible and no major serious side effects have been reported so far. One of the risks of CNI withdrawal is an increased risk of acute allograft rejection. Therefore renal function and trough levels of the immune suppressive drugs will be monitored frequently. In addition, the type of induction therapy (alemtuzumab) and the timing of CNI withdrawal (>6 weeks) are chosen to minimize risks for allograft rejection. We think that early clinical results, which suggest beneficial effect from MSC administration for patients after renal transplantation and the expected limited possibility on adverse side effects justify participation in this study.

## Discussion

The great potential for MSC therapy to become a new tool after renal transplantation as immune suppressive and reparative treatment is strengthened by positive preclinical results, the ease of isolation and expansion of MSCs and encouraging preliminary trials. Since current immunosuppressive drugs cannot be withheld from patients receiving MSC treatment after renal transplantation, it is of importance that an optimal concurrent immunosuppressive regimen is chosen with minimal side effects. The objective of the current study is to implement MSC treatment in combination with everolimus to facilitate CNI withdrawal with the aim to preserve renal function and structure.

Since the early 1980, the standard approach to immunosuppression in transplant recipients has involved the use of CNI such as cyclosporine (CsA) and tacrolimus. Most centres nowadays use a regimen of basiliximab, mycophenolate mofetil (MMF), and corticosteroids in combination with low-dose tacrolimus, based on the Symphony trial [44]. However, immune suppressive drug treatment is becoming more and more individualized. CNI nephrotoxicity may account for the paradox that the reduction or abolition of early episodes of acute rejection has not resulted in commensurate improvements in the long-term outcome [1,45]. The damage by CNI is not reversed by mild-to-moderate reductions in the dose of these agents [45]. However, the optimal treatment strategy to reduce or eliminate CNI therapy at an early time point after transplantation, without compromising efficacy, is unclear. In this perspective MSCs might facilitate CNI withdrawal. In our protocol, we have chosen for the combination of MSCs with everolimus, prednisolone and alemtuzumab. The combination of an mTOR inhibitor and MSCs might be a potential promising strategy since there is experimental evidence that this combination is tolerogenic [2,9,29] and capable of increasing regulatory immune cells [29]. Of importance, complete avoidance and replacement of a CNI by everolimus in de novo transplant recipients is not justified, since this strategy previously resulted in unacceptable high acute rejection rates even with induction therapy [46]. Both the Caesar and the Symphony study showed that reduced CNI-dosing, as opposed to full dose CNI early after transplantation, are equally efficacious in preventing acute rejection but only marginally improved renal function parameters [44,47]. The CONVERT and the ASCERTAIN study were initiated to replace CNIs by an mTOR inhibitor at a late time-point i.e., 3,2 and 5,6 years respectively, after transplantation [48,49]. This strategy proved to be safe but again only minor improvements in renal function parameters were found and if so, predominantly in patients with still preserved renal function. Contrastingly, in the Zeus study, renal allograft recipients were converted from CNI to everolimus at 4–5 months after transplantation to triple drug regimen with mycophenolate and steroids and reported significantly better renal function up to 3 years after CNI-elimination [50,51]. In our study, CNI will be withdrawn at an earlier time point, namely at 6 weeks after transplantation. To decrease the risk for acute rejections, MSCs will be infused at the time of CNI withdrawal. In addition, alemtuzumab will be used as induction treatment. This induction treatment has been shown to be superior to traditional antibodies in preventing acute rejection. A recent study demonstrated that alemtuzumab-based induction therapy followed by reduced CNI and mycophenolate exposure and steroid avoidance reduced the risk of biopsy-proven acute rejection compared with standard basiliximab in a broad range of patients receiving a kidney transplant [52].

One of the important issues in the MSC-based clinical trials is defining endpoints, as this is the measure of trial failure or success. However, this is very challenging in patients after solid organ transplantation, where an increased risk for infections and malignancies already exists and where there is a lack of validated surrogate markers of disease. The therapeutic aim of MSC-based trials in renal transplant recipients is to induce immune suppression and repair of damaged tissue but without the risk of inducing tumours, infections, or unwanted tissue development, and obviously without increasing allograft rejection rates and diminishing allograft survival. Therefore both safety and efficacy measures have to be addressed. Efficacy markers in our trial include laboratory studies to determine possible development of donor-specific immunosuppression and histopathologic evaluation of renal tissue to evaluate both inflammation and fibrosis before and after the treatment. The primary endpoint in our study is the comparison of fibrosis by quantitative SR scoring of MSC treated and untreated groups at 6 months compared to 4 weeks post-transplant. Once established, interstitial fibrosis and arteriolar hyalinosis lead to progressive glomerulosclerosis over the subsequent years. In earlier studies nephrotoxicity, implicated in late ongoing injury, has been shown to be almost universal at 10 years, even in grafts with excellent early histologic findings. By 10 years, severe chronic allograft nephropathy was present in 58.4% of patients, with sclerosis in 37.3% of glomeruli. Tubulointerstitial and glomerular damage, once established, was irreversible, resulting in declining renal function and graft failure [38,53-56]. Functional studies underestimate the extent of allograft disease as supported by longitudinal studies of protocol renal biopsies showing histologic features of IF/TA in well-functioning grafts [38,39,56-58]. Therefore, early histologic detection of IF/TA has been suggested to be helpful in predicting the risk for subsequent loss of function and time to graft failure and to estimate the efficacy of therapeutic measures. Semiquantitative grading systems such as Banff scores have a wide inter-observer variation that makes comparison across centers inaccurate. In contrast, computerized image analysis of fractional interstitial fibrosis of SR-stained biopsies has been shown to be a valid and reproducible method to measure the degree of fibrosis [40,41,43,59,60]. SR dye is specific for collagen types I and III, which represent respectively 80 and 20% of total collagen synthesized by fibroblasts and thereby important components of renal matrix. Quantification of renal interstitial volume assessed by SR nonpolarized technology has been validated and correlated significantly with GFR as measured by iothalamate clearance in cases of established chronic allograft nephropathy [59]. In addition, in 2011 morphometric and visual evaluation of fibrosis by various techniques was compared and Collagen III, SR unpolarized had strongest correlations, greatest dynamic range and the best correlation with estimated GFR [61].

As secondary endpoints safety measures will be included. An important safety issue includes direct toxicity related to the infusions. Although, to date, no toxicity has been observed during intravenous MSC infusion [11], a possible side-effect of MSC transfusion could be a transfusion reaction (e.g. allergic reaction, fever and hypotension). Another possible side effect could be an increased risk of infection; therefore all donors are thoroughly screened before BM aspiration. In addition, one cannot rule out that a renal transplant recipient receiving MSCs might have a decrease of renal function. In addition, the change in immune suppressive regimen might increase the incidence of acute rejection.

Other potential risks which should be considered when using MSCs for clinical applications include risk for malignancies and opportunistic infections. So far, in clinical trials using MSCs no malignancies have been reported. However most trials have a short follow up and we are still awaiting reports on long-term effects [20,62,63]. Moreover, in many trials patients with a poor prognosis are included where MSC-related side effects might be obscured. In addition, due to the concomitant use of immunosuppressive medications, patients who received a renal transplant are already at enhanced risk of malignancies.

The risk of over immunosuppression, which may lead to opportunistic infections, has been studied in a few reports and will be closely monitored in our trial as well. In a study by Tan et al. on patients undergoing renal transplantation [32], the use of autologous MSCs instead of anti-IL-2 receptor antibody induction therapy resulted in decreased opportunistic infections (including CMV infections). However, CMV donor recipient status was negative in 151 of 154 patients, which might have caused the low incidence of CMV infections in their population. In our previous trial, 3 out of 6 renal recipients developed opportunistic infections [34] and also MSC co-infusion after haematopoietic stem cell transplantation (HSCT) caused a higher 1-year incidence of (particularly fungal) infections [64]. It is clear that frequent and accurate monitoring of infectious complications and also monitoring of the level of immune suppression remains essential.

A number of factors may adversely affect renal structure and function, including ongoing alloimmune injury by cellular and immune reactions. It is therefore of major importance that the immune status is accurately measured in our trial population. Our current protocol will measure DSA at several time points and will encompass a robust immune monitoring strategy, developed by the ONE study consortium which includes procedures for whole blood leukocyte subset profiling by flow cytometry [36]. Our hypothesis is that MSCs induce immune suppression by decreasing the amount of effector cells and increasing the number of regulatory cells. In our previous trial, we have shown that patients receiving MSCs demonstrated a profound reduction in proliferation of PBMC 12 weeks after MSC infusion upon stimulation with donor PBMCs, but there were no consistent changes in immune profiles. In the pilot study by Perico et al. MSC treatment allowed increased numbers of T regs in the peripheral blood and control of memory CD8^+^T cell function [30]. In both studies the amount of included patients was small and it is therefore difficult to draw firm conclusions. In addition, both studies were set up as safety and feasibility studies. In the current trial we anticipate that we can make meaningful comparisons of leukocyte subset profiling in MSC treated and untreated groups.

Cardiovascular disease exerts a high burden in terms of morbidity and mortality in renal recipients. The annual risk of a cardiovascular event is up to 50-fold higher for a renal recipient compared to the general population [65-67] and over a third of all deaths following kidney transplantation are caused by cardiovascular disease [68,69]. Several studies nowadays focus on the proposed cardio protective effects of everolimus in transplant recipients. In heart transplantation everolimus was significantly more efficacious than mycophenolate mofetil in preventing cardiac allograft vasculopathy (CAV) as measured by intravascular ultrasound (IVUS) among heart-transplant recipients after 1 year [70]. Interestingly, this process might be regulated by T regs, which are expanded after treatment of an mTOR inhibitor [71]. However, mTor inhibitors are also capable to increase the frequency of antigen-specific CD8^+^T cells that differentiated into the memory lineage, thus adequate immune monitoring is of major importance [72]. As MSCs have also been proposed to exert reparative properties in cardiovascular disease, the combination of MSCs and everolimus might be an opportunity to reduce the toll of cardiovascular disease following kidney transplantation.

Taken together, we hypothesize that infusion of MSCs enables CNI withdrawal and provides a novel treatment option for renal recipients with a profound effect on the fibrosis reaction and less side effects than existing immunosuppressive therapies. A positive outcome from MSCs in terms of safety and preservation of renal function and structure would implicate a major advancement for renal transplant recipients.

## Abbreviations

BM: Bone marrow
BPAR: Biopsy proven acute rejection
CAV: Cardiac allograft vasculopathy
CMV: Cytomegalovirus
CNI: Calcineurin inhibitors
CsA: Cyclosporine
DSA: Donor specific antibody
IF/TA: Interstitial fibrosis and tubular atrophy
DSMB: Data safety monitoring board
GFR: Glomerular filtration rate
HSCT: Hematopoietic stem cell transplantation
IVUS: Intravascular ultrasound
I/R: Ischemia reperfusion
MMF: Mycophenolate mofetil
MSCs: Mesenchymal stromal cells
MTOR: Mammalian target of rapamycin
SAE: Serious adverse event
SCR: Subclinical rejection
SR: Sirius red
Tol-DCs: Tolerogenic dendritic cells
